# Early Breast Cancer: Could Combined LOCalizer^TM^ and Ultrasound Localization Replace the Metallic Wire? A Multicentric Study

**DOI:** 10.3390/jcm13061713

**Published:** 2024-03-16

**Authors:** Simona Parisi, Claudio Gambardella, Antonio Santoriello, Roberto Ruggiero, Francesco Iovino, Francesca Fisone, Federico Maria Mongardini, Francesco Saverio Lucido, Salvatore Tolone, Ludovico Docimo

**Affiliations:** 1 Department of Advanced Since and Surgery, General, Mini-Invasive, Oncological and Obesity Surgery, Luigi Vanvitelli University of Campania, 80131 Naples, Italy; claudio.gambardella2@unicampania.it (C.G.); roberto.ruggiero@unicampania.it (R.R.); francescosaverio.lucido@unicampania.it (F.S.L.); salvatore.tolone@unicampania.it (S.T.); ludovico.docimo@unicampania.it (L.D.); 2 Luigi Cobellis Hospital of Salerno, 84078 Vallo della Lucania, Italy; antoniosantoriello2@gmail.com; 3 Department of Traslational Medical Sciences, Luigi Vanvitelli University of Campania, 80131 Naples, Italy; francesco.iovino@unicampania.it

**Keywords:** breast cancer, LOCalizer, wire-guided localization, ultrasound localization

## Abstract

**Background**: Breast localization plays a key role in early breast cancer (BC) surgery. The current gold standard is wire-guided localization (WGL), despite the known disadvantages. The patients often experience anxiety and discomfort due the metallic wire placed in the breast, and surgeons are compelled to perform the surgery on the same day as the radiological release of the wire to prevent migrations or breakages. Various wireless systems have been proposed as alternative to WGL. LOCalizer^TM^ offers the advantage of providing the exact distance from the marker called Tag. The combined technique using LOCalizer^TM^ and US allows for determining the distance from the BC margin, a critical surgical goal for oncological radicality. **Methods**: Patients referred for breast surgery to two Italian hospitals were enrolled and divided into two groups: Group A, including patients undergoing a combined approach, and group B, including patients treated with conventional WGL. **Results**: The combined approach with LOCalizer^TM^ and US was associated with better outcomes in terms of oncological radicality, cosmetic results, and patients’ satisfaction. **Conclusions**: In the current study, LOCalizer^TM^ associated with US could be considered an excellent approach for localizing non-palpable BC. Further larger comparative studies are needed to address this issue.

## 1. Introduction

Breast cancer (BC) is the most diagnosed tumor in females, followed by colon-rectal cancer, lung cancer, and thyroid cancer [1,2]. Worldwide, there were about 2.1 million newly diagnosed cases of breast cancer in 2018, and its incidence has increased since the introduction of mammography screening and the availability of innovative diagnostic technologies [3]. These factors have contributed enormously to the improvement of the detection rate of non-palpable breast lesions, which are mainly candidates for ultrasound-guided core biopsies or stereotactic vacuum biopsies, while surgical excisions are performed increasingly rarely [4]. On the other hand, the key role of breast surgery as the primary treatment has increased, and the removal of early breast cancers (BCs) is recommended by international guidelines [5]. The main goal of neoplastic breast surgery is certainly oncological radicality, and the current state of the art was validated in the Saint Gallen Consensus in 2015 as “absence of ink on tumor” for invasive BC and a distance of 2 mm (mm) for in situ BC from the specimen’s margins [6]. Therefore, healthy tissue sacrifice is not necessary, and minimally invasive approaches are encouraged in order to achieve good cosmetic results. Wire-guided localization (WGL) represents the current gold standard technique for non-palpable breast lesions. The hook-wires positioned for the localization of the lump can move forward in the breast and are not repositionable; conversely, the spiral and J needles are more manageable. WGL is compatible with mammography, ultrasound (US), and magnetic resonance imaging (MRI). The main advantages include the familiarity and availability of the technique and the use of cheap materials. Furthermore, multiple wires can set up the bracket localization in the case of multifocal BC. However, the technique is related to well-known disadvantages such as limited accuracy in dense breast tissue and deep lesions, elevated discomfort for the patients, dislodgement, pneumothorax, breakage, and infection. Moreover, since the device cannot remain in place for more than 24 h, it is usually necessary to perform the wire placement on the same date as the surgery, forcing a tight and often uncomfortable coordination between radiologists and surgeons. Another important limitation is that the entry point of the wire does not always correspond to the incision site and only the outer segment is visible, while the orientation and length of the wire are assessed only on a control image. Several wireless localizing systems have been proposed to improve breast localization in order to overcome the limitations of WGL [7]. LOCalizer^TM^ is a composite device including the following elements: a Tag, an RFID chip; an Applicator, a 12-Gauge needle; a Reader, a console with a display; and a Pencil, a very handy surgical probe able to activate the Tag into the lesion and guide the surgeon looking for the marked lesion. The main features of the system are the following: each Tag is associated with a unique identification number, which is very useful in the case of two or more lesions to excise; the device can indicate the exact distance in mm between the Tag and Pencil [8].

Considering the latter peculiarity, Parisi et al. proposed a combined localizing technique with ultrasound and LOCalizer^TM^ based on the possibility to calculate according to difference the exact distance from the edges of the lesion. Satisfying results regarding oncologic radicality and cosmetic outcomes were described [9]. Therefore, the exclusive use of LOCalizer^TM^ was compared to the combined technique, and we report encouraging advantages for the latter [10].

The current study aims to evaluate the accuracy and efficacy of the combined use of LOCalizer^TM^ and US compared with WGL for early BC localization in terms of oncological radicality and the satisfaction of clinicians and patients. 

## 2. Patient and Methods

### 2.1. Study Design

This study is reported following the STROBE statement for cohort studies [11]. A retrospective multicenter study was conducted to compare the efficacy of the combined use of LOCalizer^TM^ and US with WGL alone in the detection of early BC. It was conducted according to the ethical principles stated in the Declaration of Helsinki. Written informed consent was obtained from all subjects. 

### 2.2. Study Setting and Study Population

From June 2020 to May 2023, all patients with breast cancer referred to the Division of General Surgery of the Teaching Hospital “L. Vanvitelli” in Naples and “L. Cobellis” Hospital in Salerno were considered for enrollment in the study. Inclusion criteria were age ≥ 18 years and the presence of early non-palpable BC (T1 according to TNM classification [12]) confirmed by a core biopsy. Exclusion criteria were patients with in situ BC and multiple microcalcifications due to the high risk of multicentric extension. 

An accurate explanation of all possible options for breast localization was performed. Patients accepting radiofrequency localization with LOCalizer^TM^ and US or WGL signed the informed consent form. All clinical data were recorded in an electronic database. Patients who received combined localization with LOCalizer^TM^ and US were considered in Group A. Patients who opted for traditional WGL were considered in Group B. All surgeries were performed by experienced breast surgeons (over 500 procedures). 

### 2.3. Combined Technique: LOCalize^TM^ and US

**Figure 1:**
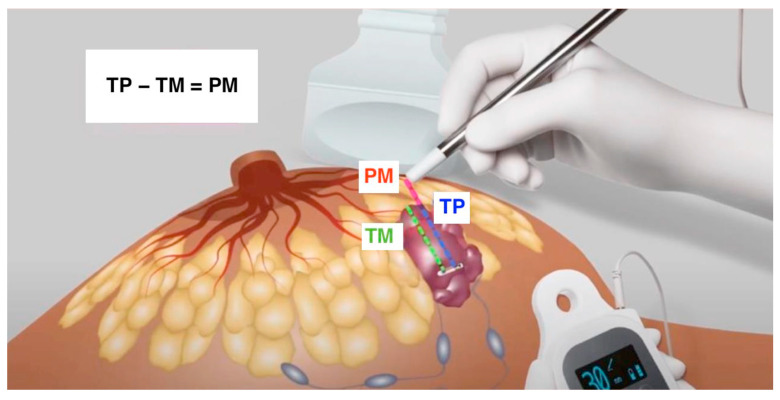
The combined technique with US and LOCalizer^TM^ can calculate the exact distance from the margins with the subtraction “TP (Tag-Pencil) − TM (Tag-Margin) = PM (Pencil-Margin)”.

Early BC localization was performed with LOCalizer^TM^ in conjunction with US. The LOCalizer^TM^ system comprises the Tag, a microchip emitting radiofrequency signals; the Pencil, a surgical probe; and the Reader, a console characterized by a display showing the exact distance in millimeters from the Pencil to the Tag and emitting an acoustic signal. The Tag was placed 10–30 days before the planned surgery, introducing the percutaneous 12-gauge Applicator under US with local anesthesia. The placement aimed to release the Tag perfectly into the lesion, but it could also be located near or only partially within the lump. For the US, a SuperSonic^®^ MACHTM 30 (Hologic, Santa Carla, CA, USA) machine with a 5–18 MHz conventional linear transducer was adopted. The sonographer (a surgeon with certified ultrasonographic intraoperative experience in more than 300 procedures) used a B-Mode scan and the option to measure the distance between two points. The US was performed just after the placement, and the following parameters, collectively named “TM (Tag-Margin)” distances were recorded: the measures from the cranial, caudal, medial, lateral, upper, and lower margins to the Tag. The reference points on the Tag and in the margins were always the midpoints for each side. When the Tag midpoint was outside the lesion, it was recorded with a minus sign. On the surgery day, US scans were performed to evaluate any possible migrations, and the TM parameters were rechecked. During the surgery, the Pencil was used to identify the Tag within the parenchyma, following the sound intensity and the distance reported on the Reader Display. This latter is defined as “TP (Tag-Pencil)”. From the cranial, caudal, medial, lateral, upper, and lower directions, the surgeons compared the recorded US measures “TM” with the millimeters reported on the display “TP”. In this way, they could determine the distance between the Pencil and the margin, named “PM (Pencil-Margin)”, through the following subtraction: “TP − TM = PM”. After the excision, they verified the absence of a signal in the residual parenchyma and the presence of the Tag in the specimen. New coordinates, TM, were measured to obtain the first immediate confirmation of oncological radicality. Furthermore, the specimen was sent to pathologists. The time consumed was calculated from the surgical incision to the sending of the specimen, including the last post-excisional US evaluation (Figure 1). 

### 2.4. Wire-Guided Localization

Early BC localization was performed with Relock Premium Duo (Vigeo s.r.l., Mantova, Italy). The wire was placed on the same day as the planned surgery, under local anesthesia. The placement aimed to allocate the point into the lesion, but it could also be located near or only partially into the lump. The control US was performed using a SuperSonic^®^ MACHTM 30 (Hologic, Santa Carla, CA, USA) machine with the same 5–18 MHz conventional linear transducer. The sonographer (a surgeon with certified ultrasonographic intraoperative experience in more than 300 procedures) used a B-Mode scan to guide the placement. 

### 2.5. Outcome Measures

The operative time was calculated in minutes from the skin cut to the early BC excision. The volume and the weight of the specimens were evaluated by the pathologists, and such parameters correlated to a mini-invasive surgical approach. The volume was estimated by measuring the three coordinates of the BC, width, length, and height in cm^3^, using the ellipsoid formula. Lumpectomy volume was calculated from the specimen’s dimensions (anterior–posterior, medial–lateral, superior–inferior) provided in the pathology report. 

The weight was obtained using the pathological report and measured on a Kern FOB-NLO balance (Kern, Balingen, Germany). Oncological radicality was assessed with a definitive pathological examination of the specimens sent to the Pathological Institute of the Hospital. According to Saint Gallen’s recommendation, when the cancer margin was not inked upon microscopic observation, the surgical radicality was reached and reported as R0 surgery on the pathological report. Clinicians’ and patients’ satisfaction were evaluated with a questionnaire 30 days after the surgery. Clinicians’ tests investigated their opinions regarding the difficulties, the choice of the incisional cut, and the reliability of the localization. The patients were asked about anxiety between the localization and the surgery, the discomfort, the pain, and the fear of delocalization. A Likert scale with a 0–10 point range was adopted. Furthermore, clinicians and patients were asked about their overall satisfaction [13]. 

### 2.6. Study Endpoints

The primary endpoint was the evaluation of oncological radicality based on the definitive pathology of specimens belonging to combined LOCalizer^TM^ and US group versus patients treated with WGL. The secondary endpoints were the assessment of clinicians’ and patients’ satisfaction with BC localization using the combined LOCalizer^TM^ and US method versus WGL. 

### 2.7. Statistical Analysis

Statistical analysis was performed via Excel 2011^®^ (Microsoft, Redmond, WA, USA) and the Graph-Pad Prism^®^ 9 program (San Diego, CA, USA). Categorical data were reported as raw numbers with percentages in parentheses. Continuous data were reported as means ± standard deviation or as medians with the range in parentheses, according to the distribution. The differences between results were analyzed using the unpaired *t*-test; if they were summarized as means, the Mann–Whitney test was used. 

## 3. Results

From June 2020 to January 2023, 216 women received diagnoses of early BC (diameter < 20 mm) at the Division of General Surgery of the Teaching Hospital “L. Vanvitelli” in Naples and “L. Cobellis” Hospital in Salerno. Only 166 of them met the inclusion criteria, were undergoing breast localization, and were considered in the current analysis. Sixty-eight women received breast localization with LOCalizer^TM^ and US and were included in Group A. Ninety-eight patients received WGL (Group B). The baseline demographic and clinical characteristics are reported in Table 1. 

The operative time, calculated from incision to excision, despite the double technique, was 14.8 ± 7.0 min for Group A compared to 13.9 ± 5.1 min for Group B, with a difference that was not statically significant (*p* = 0.720). The mean specimen volume was 14.4 ± 6.9 cm^3^ for Group A and 20.6 ± 6.5 cm^3^ for Group B, while the mean specimen weights were 19.2 ± 4.8 g and 24.0 ± 5.1 g, respectively (*p* = 0.003 and *p* = 0.002) (Table 2).

### 3.1. Primary Outcome

The primary aim of this clinical study was to evaluate oncologic radicality after a definitive pathological examination, following the Saint Gallen indication (“no ink on the tumor”). This criterion was achieved for 68 patients (100%) in Group A and 92 patients (93.9%) in Group B, with a non-statically significant difference (*p* = 0.004). 

### 3.2. Secondary Outcome

Patient and clinician satisfaction were assessed through a survey conducted 30 days after the surgery, when cosmetic results were definitive and histological reports were available. Patients expressed high satisfaction with the combined technique due to its less invasive nature. They were questioned about anxiety, discomfort, and fear of delocalization. The values were recorded according the Likert scale and are reported in Table 2. The differences appeared to be statistically significant in favor of the combined approach for all the items. No significant differences were reported regarding pain associated with the placement of Tags or wires, which was performed under local anesthesia. Better cosmetic results were reported with combined localization (8.6 ± 0.8 vs. 7.0 ± 0.7, respectively, for Groups A and B, *p* = 0.001). Furthermore, surgeons considered LOCalizer^TM^ and US localization to be safer and more accurate than the standard use of metallic wires. No cases of recurrence or mortality were recorded after a mean follow-up of 18 months. 

## 4. Discussion

In the past, breast localization was necessary for excisional biopsies and conservative surgery. WGL has been the preferred method for many breast units worldwide over the last thirty years. It involves inserting a metallic wire into the breast tissue to indicate the occult lesion, typically performed under mammographic or ultrasound guidance. It can also be performed under magnetic resonance imaging, but this approach is reserved for selected cases due to the associated high costs [8,9,10,11,12,13,14,15]. However, WGL has several limitations, including migration issues (up to 3% of patients), patient discomfort, and the risks of wire fracture during the transfer from the radiology department to the operating room. To minimize these issues, wire placement is often performed on the same day as surgery, leading to logistical challenges and various adverse events such as pneumothorax, bleeding, and infections [16]. One significant limitation of WGL is that the incisional cut and the point of wire placement on the skin are often different, requiring the surgeon to modify the incision. Despite efforts to optimize this technique [17], patient stress remains a challenge. Clear margins obtained with WGL are reported to be 70.8% to 87.4% [18]. While WGL remains the standard method for breast localization, new wireless technologies have been proposed. Recently, the FDA approved a new device, LOCalizer^TM^, aiming to improve the identification of non-palpable breast lesions. It involves the positioning of a Tag recognized by a dedicated surgical probe called the Pencil. While the preliminary results compared to conventional WGL were positive, limited data on its use have been reported. In a 2019 study by McGugin et al., LOCalizer^TM^ identification was compared to that of WGL in 503 procedures. All intended targets were successfully removed, and the specimen volumes were similar (*p* = 0.560 and 0.494), as were the operative times (*p* = 0.152 and 0.158). Re-excision rates were comparable by the surgical procedure (*p* = 0.615), surgical indication (DCIS *p* = 0.145; invasive carcinoma *p* = 0.759), and confirmed by multivariable analysis (OR 0.754, 95% CI 0.392–1.450; *p* = 0.397) [19]. Several studies have assessed the feasibility of the LOCalizer^TM^ system, focusing on various aspects such as the distance from the Tag in mm on the console; the unique code for each Tag, which is particularly useful for multiple localizations; and its potential use for axillary tailored dissections (TAD) [20]. In a previous study, a combined technique with US and the LOCalizer^TM^ system was utilized, showing encouraging results. The choice of combining both techniques (US and LOCalizer^TM^ system) arises from the ability to dynamically assess the distance from the margin of the lesion during the surgical procedure, rather than relying on the Repere, which could be placed in an eccentric position or even outside the measurement. Monitoring distances from the margins could also be achieved with mammography; however, this technique does not allow for real-time assessment during excision.

To the best of our knowledge, the current study is the first to compare the surgical and oncological outcomes of the combined LOCalizer^TM^ and US localization versus WGL alone for non-palpable BC. The primary endpoint was to evaluate oncologic radicality, and the combined method appeared to be safer and more accurate than WGL (100% vs. 93.4% cancer free margins, respectively). The results of the combined technique are even more encouraging than outcomes reported in literature regarding the exclusive use of the radiofrequency system. Perhaps the use of US and the evaluation of distances from the margins can improve surgeons’ orientation. Law et al. reported a re-operation rate of 6% in their study, assessing the adequacy of invasive and in situ breast carcinoma margins in radioactive seed- and wire-guided localization lumpectomies [21]. Regarding the radiofrequency system, different rates of R0 surgery have been described. Christenhusz et al. found clear resection margins in 92.7% of the cases (89 out of 96 patients), while Lamb et al. reported that 15.1% of surgeries had positive or close surgical margins requiring re-excision [22,23]. In 2021, a French group published a study protocol aiming to demonstrate the superiority of the RFID technique in terms of patient tolerance compared to the gold standard (hook wire). The study involved patients filling out a satisfaction questionnaire during two steps: during the placement of the device (RFID tag or hook wire) and during the postoperative consultation at one month. Radiologists and surgeons were also required to complete a questionnaire to evaluate the localization technique after the localization and surgery procedures, respectively [24]. In the current study, higher clinician and patient satisfaction was significantly reported for the combined approach for all the domains analyzed (*p* < 0.001). Regarding the patients’ satisfaction, this could possibly be related to the lower specimen volume (14.4 ± 6.9 cm^3^ vs. 20.6 ± 6.5 cm^3^, *p* = 0.003) and the lower specimen weight (19.2 ± 4.8 g vs. 24.0 ± 5.1 g, *p* = 0.002) in patients undergoing the combined approach, guaranteeing, as mentioned previously, a higher oncological radicality rate. Surgical excision of a non-palpable breast lesion requires a localization step. Among the available techniques, WGL is the most commonly used. Other techniques have been developed in the last two decades with the aim of improving outcomes and logistics. Intraoperative ultrasound is associated with significantly higher negative margin rates, and radioactive techniques are non-inferior to WGL. 

One of the limitations is certainly represented by the cost of the localizer system, which is around EUR 500 per procedure. 

Additional limitations of the study include the following: The volumes of the excised histological specimens depend on the approximation derived from the use of the ellipsoid formula. Furthermore, the retrospective nature and patient enrollment were conducted based on the participants’ personal choices and preferences, allowing them to choose either technique. The absence of randomization may have introduced bias. 

Large studies with an additional focus on the patients’, surgeons’, and radiologists’ preference are necessary. This is the rationale for the MELODY (NCT05559411) study and to enable the standardization of outcome measures for future studies [25].

## 5. Conclusions

WGL represented the gold standard for the localization and removal of non-palpable breast lesions for more than a century. Numerous limitations of the technique from a patient’s as well as a surgeon’s point of view have put this standard into question for almost two decades. In the current study, LOCalizer^TM^ with US has emerged as the preferred option for localizing non-palpable BC. It has facilitated minimally invasive resection with excellent oncological outcomes. It is worth commenting on the data indicating higher patient satisfaction with the combined technique, whereas wire-guided localization (WGL) was associated with elevated stress due to the presence of the wire in the breast. This observation underscores the importance of also considering the patients’ experiences in the establishment of a future gold standard for breast localization. Larger comparative studies are needed in order to address this issue.

## Figures and Tables

**Table 1:** Baseline demographic and clinical characteristics.

	Group A 68 Patients	Group B 98 Patients	*p*
Age (years) °	56.8 ± 9.4	57.8 ± 12.4	*p* = 0.840
BMI °	29.6 ± 5.9	26.6 ± 5.8	*p* = 0.220
Staging			
pT1a	29 (42.6%)	39 (39.8%)	*p* = 0.680
pT1b	23 (33.8%)	33 (33.7%)	*p* = 0.980
pT1c	16 (23.6%)	26 (26.5%)	*p* = 0.980
Breast Side			
Right	37 (54.4%)	50 (51.0%)	*p* = 0.630
Left	31 (45.6%)	48 (49.0%)	*p* = 0.810
Breast region			
Upper Lateral Quadrant	31 (45.6%)	38 (38.8%)	*p* = 0.100
Upper Medial Quadrant	15 (22.1%)	34 (34.8%)	*p* = 0.004 *
Lower Lateral Quadrant	13 (19.1%)	13 (13.2%)	*p* = 0.250
Lower Medial Quadrant	9 (13.2%)	13 (13.2%)	*p* = 1.000

Data are reported as number of cases or median ± standard deviation °. BMI (body mass index). * Statistically significant.

**Table 2:** Outcomes.

	Group A 68 Patients	Group B 98 Patients	*p*
Oncologic radicality at definitive pathological exam °	68 (100%)	92 (93.9%)	*p* = 0.004 *
Operative time (minutes) °	14.8 ± 7.0	13.9 ± 5.1	*p* = 0.720
Specimen volume (cm^3^) °	14.4 ± 6.9	20.6 ± 6.5	*p* = 0.003 *
Specimen weight (g) °	19.2 ± 4.8	24.0 ± 5.1	*p* = 0.002 *
Patients’ Satisfaction			
-anxiety	8.6 ± 0.5	7.8 ± 0.9	*p* = 0.001 *
-discomfort	5.1 ± 1.1	6.5 ± 1.4	*p* = 0.001 *
-pain	5.5 ± 1.2	7.0 ± 1.4	*p* = 0.001 *
-fear for dislocation	6.4 ± 1.4	6.5 ± 1.2	*p* = 0.890
-cosmetic results	2.8 ± 1.1	6.9 ± 0.9	*p* = 0.001 *
(Likert) °	−8.6 ± 0.8	7.0 ± 0.7	*p* = 0.001 *
Clinicians’ Satisfaction (Likert) °	9.7	7.8	0.003 *
Mortality	0	0	*p* = 1.00
Recurrence	0	0	*p* = 1.00

Data are reported as number of cases or median ± standard deviation °. * Statistically significant.

## Data Availability

Data are available on the hospital’s electronic database.
